# Neutralizing antibodies against human T cell leukemia virus type-I (HTLV-1) eradicate HTLV-1 in combination with autologous peripheral blood mononuclear cells via antibody-dependent cellular cytotoxicity while preventing new infection

**DOI:** 10.1186/1742-4690-11-S1-O39

**Published:** 2014-01-07

**Authors:** Yuetsu Tanaka, Yoshiaki Takahashi, Akira Kodama, Reiko Tanaka, Mineki Saito

**Affiliations:** 1 Department of Immunology, Graduate School of Medicine, University of the Ryukyus, Nishihara, Okinawa, Japan; 2 Department of Microbiology, Kawasaki Medical School, Kurashiki, Okayama, Japan

## 

In order to establish a basis for vaccine development against human T cell leukemia virus type-I (HTLV-1), we have evaluated the roles of anti-HTLV-1 neutralizing antibodies using a rat monoclonal antibody (mAb) against HTLV-1 envelope gp46 (LAT-27) and human polyclonal IgG purified from sera of HTLV-1-associated myelopathy (HAM) patients (HAM-IgG). LAT-27 and HAM-IgG completely blocked the HTLV-1-mediated syncytium formation and T-cell transformation in vitro. Interestingly, when these antibodies were added to the cultures of CD8+ T cell-depleted peripheral blood mononuclear cells (PBMCs) from HAM patients, proliferation of Tax-expressing T cells and HTLV-1 p24 production were blocked. In addition, co-culture of HTLV-1-immortalized T cells with autologous PBMCs in the presence of LAT-27 or HAM-IgG, but not F(ab)’2 fragment of LAT-27 or the other non-neutralizing anti-gp46 mAbs, resulted in eradication of Tax-expressing cells and the p24 production. A ^51^Cr release assay for 24 hours showed a significant killing of HTLV-1-infected T cells by autologous PBMCs in the presence of LAT-27 or HAM-IgG, but not F(ab)’2 fragment of LAT-27, in which depletion of CD16+ cells from the effector PBMCs significantly reduced the killing activity. Altogether, the present data demonstrated for the first time that anti-HTLV-1 gp46 neutralizing antibodies are capable of not only preventing new infection but also eliminating HTLV-1-infected cells in the presence of autologous PBMCs mainly via an antibody-dependent cellular cytotoxicity (ADCC) in vitro. Thus, a vaccine candidate that can elicit or boost anti-gp46 neutralizing antibody response may have a potential for prevention and therapy against HTLV-1 infection.

